# Enriched biodiversity data as a resource and service

**DOI:** 10.3897/BDJ.2.e1125

**Published:** 2014-06-16

**Authors:** Rutger Aldo Vos, Jordan Valkov Biserkov, Bachir Balech, Niall Beard, Matthew Blissett, Christian Brenninkmeijer, Tom van Dooren, David Eades, George Gosline, Quentin John Groom, Thomas D. Hamann, Hannes Hettling, Robert Hoehndorf, Ayco Holleman, Peter Hovenkamp, Patricia Kelbert, David King, Don Kirkup, Youri Lammers, Thibaut DeMeulemeester, Daniel Mietchen, Jeremy A. Miller, Ross Mounce, Nicola Nicolson, Rod Page, Aleksandra Pawlik, Serrano Pereira, Lyubomir Penev, Kevin Richards, Guido Sautter, David Peter Shorthouse, Marko Tähtinen, Claus Weiland, Alan R. Williams, Soraya Sierra

**Affiliations:** † Naturalis Biodiversity Center, Leiden, Netherlands; ‡ Pensoft Publishers, Sofia, Bulgaria; § Institute of Biomembranes and Bioenergetics, National Research Council, Bari, Italy; | University of Manchester, Manchester, United Kingdom; ¶ Royal Botanic Gardens, Kew, United Kingdom; # The Illinois Natural History Survey, Champaign, United States of America; †† Agentschap Plantentuin Meise, Meise, Belgium; ‡‡ Aberystwyth University, Aberystwyth, United Kingdom; §§ Botanic Garden and Botanical Museum Berlin-Dahlem, Freie Universität Berlin, Berlin, Germany; || The Open University, Milton Keynes, United Kingdom; ¶¶ Museum für Naturkunde, Berlin, Germany; ## University of Bath, Bath, United Kingdom; ††† University Of Glasgow, Glasgow, United Kingdom; ‡‡‡ Software Sustainability Institute, Manchester, United Kingdom; §§§ Biodiversity Informatics Consultant, Christchurch, New Zealand; ||| Plazi, Bern, Switzerland; ¶¶¶ Université de Montréal Biodiversity Centre, Montréal, Canada; ### University of Eastern Finland, Espoo, Finland; †††† Biodiversity and Climate Research Centre, Senckenberg Gesellschaft für Naturforschung, Frankfurt, Germany

**Keywords:** Biodiversity informatics, Data enrichment, Hackathon, Intelligent openness, Linked data, Open source, Software, Semantic Web, Taxonomy, Web services

## Abstract

**Background:** Recent years have seen a surge in projects that produce large volumes of structured, machine-readable biodiversity data. To make these data amenable to processing by generic, open source “data enrichment” workflows, they are increasingly being represented in a variety of standards-compliant interchange formats. Here, we report on an initiative in which software developers and taxonomists came together to address the challenges and highlight the opportunities in the enrichment of such biodiversity data by engaging in intensive, collaborative software development: The Biodiversity Data Enrichment Hackathon.

**Results:** The hackathon brought together 37 participants (including developers and taxonomists, i.e. scientific professionals that gather, identify, name and classify species) from 10 countries: Belgium, Bulgaria, Canada, Finland, Germany, Italy, the Netherlands, New Zealand, the UK, and the US. The participants brought expertise in processing structured data, text mining, development of ontologies, digital identification keys, geographic information systems, niche modeling, natural language processing, provenance annotation, semantic integration, taxonomic name resolution, web service interfaces, workflow tools and visualisation. Most use cases and exemplar data were provided by taxonomists.

One goal of the meeting was to facilitate re-use and enhancement of biodiversity knowledge by a broad range of stakeholders, such as taxonomists, systematists, ecologists, niche modelers, informaticians and ontologists. The suggested use cases resulted in nine breakout groups addressing three main themes: i) mobilising heritage biodiversity knowledge; ii) formalising and linking concepts; and iii) addressing interoperability between service platforms. Another goal was to further foster a community of experts in biodiversity informatics and to build human links between research projects and institutions, in response to recent calls to further such integration in this research domain.

**Conclusions:** Beyond deriving prototype solutions for each use case, areas of inadequacy were discussed and are being pursued further. It was striking how many possible applications for biodiversity data there were and how quickly solutions could be put together when the normal constraints to collaboration were broken down for a week. Conversely, mobilising biodiversity knowledge from their silos in heritage literature and natural history collections will continue to require formalisation of the concepts (and the links between them) that define the research domain, as well as increased interoperability between the software platforms that operate on these concepts.

## Introduction

The Royal Society report on “*Science as an Open Enterprise”* recommends that “*scientists should communicate the data they collect and the models they create, to allow free and open access, and in ways that are intelligible, accessible and usable for other specialists in the same or linked fields wherever they are in the world*” (Boulton et al. 2012). A recent, authoritative appraisal of the biodiversity informatics domain ratified these recommendations (Hardisty et al. 2013), and recent years have seen a surge in projects that produce large volumes of open biodiversity data. Some examples of these include species descriptions and occurrence data as extracted from digitised biodiversity literature, including biotas (faunas, floras, mycotas, etc.); comparative morphological and molecular data that forms the basis for phylogenetic inference and phyloinformatics; and data available from digitised collection specimens held by herbaria and natural history museums. These data can be represented in a variety of standardised, machine-readable formats, which has opened up a wealth of opportunities for data integration and “data enrichment”, i.e. the general process to refine, enhance or otherwise improve raw data.

In response to these developments, the *pro-iBiosphere* project organised over the last two years (2012 - 2014) a series of workshops, meetings and pilot projects addressing technical, interoperability, legal and sustainability issues in the European biodiversity informatics domain. These activities resulted in findings and outcomes pertaining to the discovery, identification, aggregation and annotation of data resources and services (Güntsch et al. 2013); recommendations for the semantic annotation and integration of biological publications (Mietchen 2014); and a strategy to improve machine-based dialogue of semantically-enhanced biodiversity information relating to taxonomic treatments (Agosti et al. 2013). The project is slated to culminate in the *Bouchout Declaration* (http://bouchoutdeclaration.org), which will set forth the principles that will enable the signatories to participate in collaborative, open, biodiversity knowledge management.

To build on these findings and to take advantage of the emerging opportunities, Naturalis Biodiversity Center (Leiden, the Netherlands) and the pro-iBiosphere project organised a “hackathon”, a type of meeting that focuses on intensive, collaborative open source software development that is gaining acceptance in the computational biology (*sensu lato*) community (Möller et al. 2013). From 17 – 21 March 2014, the “Biodiversity Data Enrichment Hackathon” took place at Naturalis. The purpose of the hackathon was to develop proofs-of-concept demonstrating data enrichment in biodiversity informatics by engaging in intensive, collaborative software development and data exploration.

The Biodiversity Data Enrichment Hackathon followed a use case-driven model, i.e. a model where effort before and during the hackathon was prioritised on the basis of compelling end user scenarios that could be enabled by the combined contributions of people that otherwise, outside of the hackathon, do not collaborate. This is an often-followed model for such events: past examples of this include the DBCLS BioHackathons (Katayama et al. 2010, Katayama et al. 2011, Katayama et al. 2013) and the NESCent phyloinformatics hackathons (Lapp et al. 2007, Stoltzfus et al. 2013). The approach is appropriate in cases where participants operate in overlapping domains but do not necessarily collaborate on the same projects or are familiar with the same code bases. A contrasting approach is sometimes taken, for example, in hackathons organised under the auspices of the Open Bioinformatics Foundation (http://www.open-bio.org/wiki/Hackathon), where the scope might be defined as the achievement of some measurable interoperability goal between various Bio* programming toolkits, i.e. a situation where all participants are (intimately) familiar with the same code bases and choose to achieve functionality that is deemed to be valuable in the long term, but not necessarily immediately visible from the end user perspective.

Prior to the hackathon, a wiki was created in which participants had the opportunity to describe their ideas for use cases and receive feedback from others (http://wiki.pro-ibiosphere.eu/?oldid=6911). During the first day of the meeting, participants briefly presented the use cases suggested (20 in total). Subsequently, during a self-organisation bazaar, the presenters of the use cases had the opportunity to interact with other participants and further discuss their ideas. Participants then selected by approval voting the use case(s) they found most interesting and where they could bring in their expertise for further collaboration. The approach is akin to Open Space Technology (Owen 1997), whose application was pioneered in the bioinformatics domain by the DBCLS BioHackathons. As a result of this process, the use cases were distilled into nine breakout groups (http://wiki.pro-ibiosphere.eu/?oldid=7030) broadly addressing i) challenges in the areas of literature and natural language processing; ii) interoperability challenges among workflow and data publishing platforms; and iii) challenges of formalising and ontologising concepts and links between them (see Table 1). In parallel to the work in these breakout groups, tutorials were organised to share knowledge on version control, Wikimedia projects and other topics. All hackathon participants had *a priori* indicated their agreement with open source licensing (specifically, either OSI-approved or CC-compliant) of all code and documentation produced at the hackathon, and so we present the tangible outcomes of these breakout groups for the benefit of the larger community.

## Results

### Mobilising legacy biodiversity knowledge

**Biodiversity data analytics**

*Members* - David King, Jeremy Miller, Serrano Pereira and Guido Sautter

*Accomplishments* - The advent of biodiversity data aggregators such as Plazi (Agosti and Egloff 2009) have made it possible to perform analytics on biodiversity data use and re-use. The initial use case pitch for this group proposed a “dashboard” with various graphs showing, for example, specimen citations, or the output of researchers or institutions, in order to assist with data analytics by being able to visualise and thereby better understand it, and assisting with its quality control. At the hackathon, the members of the breakout group addressed this challenge from two ends: on the server side, GoldenGATE was enhanced with a search facility that outputs selected search predicates as structured data in various views; on the web browser (i.e. client) side, this structured data was consumed by the jQuery JavaScript framework (http://jquery.com/) in conjunction with the plugins jqPlot (http://www.jqplot.com/) and jVectorMap (http://jvectormap.com/) for data visualisation. The tangible outcomes of this effort, once stable, will be deployed on the Plazi server.

*Code repository* - https://github.com/Dauvit/Data_enrichment

*Demo* - http://plazi.cs.umb.edu/GgServer/srsStats

**OCR correction**

*Members* - Roderic Page, Kevin Richards, David Shorthouse and Marko Tähtinen

*Accomplishments* - The Biodiversity Heritage Library (BHL) is a collaborative effort of natural history and botanical libraries to digitise their physical medium literature and make it available as open access publications, forming a “biodiversity commons”. The effort has resulted in large volumes of digital documents obtained by optical character recognition (OCR) of scanned, physical documents. Although the OCR quality is generally very high, errors that require human intervention to correct them are unavoidable. This breakout group addressed this challenge by developing a collaborative platform where authenticated users can correct OCR documents rendered on webpages (via DjVu XML format, http://djvu.org), with the provenance of the edits recorded in the margin of the document. The facility is “intelligent” in that it attempts to detect patterns in OCR errors (e.g. ü is recognized as ii) in order to suggest subsequent corrections to the same class of errors. In addition, the facility cross-references taxonomic names against web services provided by GlobalNames (http://globalnames.org) to normalise these and correct them consistently throughout a document. The participants of the breakout group have communicated with BHL to assess how this code might be incorporated into their web presence. There are also plans to incorporate the code into a future release of BioStor (http://biostor.org, Page 2011).

*Code repository* - https://github.com/rdmpage/ocr-correction

*Demo* - http://bionames.org/~rpage/ocr-correction/index.php

**Open access images**

*Members* - Youri Lammers and Ross Mounce

*Accomplishments* - As public access biodiversity literature is growing in volume, so too are the graphs and pictures embedded in these publications. Unfortunately, these images are “buried” in that up till now they could only be located within their publication. To mobilise and expose these images, this breakout group developed a pipeline that extracts embedded images from open access publications (as a proof of concept this was done by harvesting PDF documents from the journal *Phytotaxa*), pre-processes them (e.g. discard spurious, small figures such as journal logos; invert image negatives) and uploads them to the social web, i.e. to the photo sharing site Flickr, with periodic notifications to the Twitter account @PhytoFigs. To date over 2000 figures have been uploaded this way. Of these, some are the very first *discoverable* images (not embedded in PDF) for those taxa to be made available on the internet under an OKD-compliant open license (http://opendefinition.org) - allowing easy re-use even for commercial purposes without the hassle of asking for permission(s). Potential scopes for follow-up include teaming up with open access journals to find a stable home for this novel method of exposing images, and harvesting and processing images of phylogenetic trees (e.g. using TreeRipper, Hughes 2011).

*Code repository* - https://github.com/rossmounce/LeidenPDFhack

*Demo* - http://www.flickr.com/photos/79472036@N07/sets/72157642597074643/

### Formalising and linking concepts

**Trait ontology**

*Members* - George Gosline, Quentin Groom, Thomas Hamann, Robert Hoehndorf and Claus Weiland

*Accomplishments* - Floristic treatments of geographic regions have a long history and have resulted in a wealth of literature. To increase the accessibility of the knowledge contained in these floras, past efforts have focused on structured markup, resulting in websites such as the Digitised Flora of Central Africa and formats such as FlorML (Hamann et al. 2014). For the hackathon, marked-up floras were available that cover a large proportion of tropical plants. These were the *Flora Malesiana, Flora Zambesiaca, Flore d’Afrique Centrale* and *Flore du Gabon*. At the hackathon, a use case was presented calling for the creation of an RDF knowledge base of plant phenotypes by extracting trait data from such digitised floras. The ensuing breakout group addressed this challenge by text mining of documents to mark up relevant concepts with terms from the Plant Ontology (PO, to normalise the inhering plant anatomical parts) and the Phenotypic Quality Ontology (PATO, to normalise both “phenotypic qualities”, such as red, serrated and small, and “traits”, such as color, shape, and size). Under this approach, phenotypes are extracted from flora descriptions as Entity/Quality (EQ) statements (Gkoutos et al. 2005). From these EQ statements, an ontology was constructed that takes into account the anatomical relationships between plant parts (as represented in PO) as well as information about traits and values (as represented in PATO, Hoehndorf et al. 2010). The resulting ontology, the Flora Phenotype Ontology (FLOPO), was deposited at NCBO Bioportal. FLOPO consists of more than 25,000 classes describing plant traits and phenotypes, and every class in FLOPO has at least one taxon annotation in one of the processed floras. Work subsequent to the hackathon has additionally yielded annotations of the floras with terms obtained from the Environment Ontology (ENVO), providing a proof of concept of an ontology-mediated knowledge base that can be brought to bear on a variety of research questions on phenotype/environment interactions, functional diversity, and community ecology. Furthermore, ongoing work aims to connect the extracted taxon names in the floras using identifiers from the International Plant Names Index (IPNI, http://ipni.org) and represent them together with their environment and FLOPO annotations as Linked Data in an RDF store. Work will also continue to support multiple languages: the current examples are in English and French, but there is a strong case for supporting other languages.

*Code repository* - https://github.com/leechuck/plantphenotypes

*Demo* - http://bioportal.bioontology.org/ontologies/FLOPO

**SWeDe**

*Members* - Bachir Balech, Niall Beard and Patricia Kelbert

*Accomplishments* - The wide adoption of the REST (Representational State Transfer) design pattern for simple, stateless web services has resulted in a proliferation of different ways in which clients need to interact with biodiversity data and computational web services. Although the adoption of the design pattern has considerably lowered the barrier for developers to implement and deploy simple services, it poses challenges to clients and end users, as the way in which they are expected to interact with these heterogeneous service interfaces is not always documented clearly. Examples of this include the various “RESTful” as well as SOAP/WSDL-based web services delivered to the BioVeL project (Vicario et al. 2012). This breakout group sought to address this issue by defining a standard for documenting such web services using XML. The resulting XML schema, SWeDe (Scientific Web-service Description), provides developers with a standard way to define metadata of their service (e.g. authorship, license, suggested citation), the inputs and outputs with the available parameters and their ranges, and usage examples. The SWeDe schema re-uses several components from the Access to Biological Collections Data (ABCD) Schema (Berendsohn 2005). In addition, the members of this breakout group have developed a rudimentary application (code named “SWeDe farmer”) to simplify generation of SWeDe documents. BiodiversityCatalogue, the registry of biodiversity web services (http://biodiversitycatalogue.org), will be adopting this standard by implementing a SWeDe parser that can periodically update catalogue entries based on the state of a service provider’s SWeDe document.

*Code repository* - https://github.com/njall/XS-SWeDe, https://github.com/njall/SWeDe-Farmer

*Demo* **** - http://swede-farmer.herokuapp.com/

**Specimen links**

*Members* - Jordan Biserkov, Matthew Blissett, George Gosline, Quentin Groom, Thomas Hamann, Ayco Holleman, Peter Hovenkamp, Nicky Nicolson, Kevin Richards and Marko Tähtinen

*Accomplishments* - This breakout group addressed several related use cases from the introductory session with the aim of better linking biodiversity data to form a navigable “knowledge graph”. As a specimen-oriented example: specimens obtained from single collection events are often distributed among herbaria, where they tend to take on an isolated life of their own, with their provenance history and annotations uncoupled from other specimens collected for the same species, even during the same collection event. This fragmentation poses challenges when integrating collection data and floristic knowledge. Software previously developed at RBG Kew to build links between data sets based on configurable text-based rules was used to link up datasets, particularly to detect duplicates between digitised herbaria - Kew (K), Meise (BR), Edinburgh (E) and Naturalis - and to create specimen level citation links from scientific papers published in *PhytoKeys*. Numerous duplicate specimens among the collections were encountered, drawing attention to a greater need for collaboration and data integration among natural history collections. The software toolkit is implemented in Java, Spring and Lucene, with a JSON format web interface conforming to the Open Refine (formerly Google Refine) reconciliation service API, and the group will follow up by open-sourcing this toolkit and the rule sets used to configure matches. Frictionless integration of collection and specimen information would permit novel ways of specifying taxon concepts, relying on the emergent properties of graphs linking specimens by conspecificity (including provenance and evidence for the assertions). This was suggested by a use case pitching a “Taxonomic Mind Mapper” to enable exploration of this method of modeling and representing taxon concepts. The application of graph database technology (Neo4J, http://neo4j.org) towards the Taxonomic Mind Mapper demonstrated an attractively low entrance barrier to linking, exploring and visualising disparate collection data. As follow-up, a publication discussing this novel way of representing taxon concepts enabled by this approach is in preparation.

*Code repository* - https://github.com/RBGKew/leiden-hackathon

*Demo* - http://gist.neo4j.org/?9684109

### Workflow and data publishing platforms

**EDIT Platform Common Data Model API**

*Members***** - Quentin Groom and Patricia Kelbert

*Accomplishments* - The Common Data Model (CDM, Berendsohn et al. 2011) consists of a database schema and application programming interface that provide a persistence and publishing mechanism for nomenclature, taxonomy, descriptive data, media, geographic information, literature, specimens, and persons. As such, instances of CDM databases provide a wealth of structured data that can be re-used in a variety of research contexts, such as in species distribution modeling. To enable such re-use, CDM databases should be enhanced with a machine-readable data harvesting service. To address this challenge, this breakout group developed a service to look up and export occurrence data (specimen, observation) from CDM instances. The lookup is based on a single Taxon UUID that can be obtained from the Name-Catalogue web service (provided by the CDM). To prevent memory overload, both on the server and on the client side, a paging system is used. The web service has been developed in the CDM library and has been committed to the CDM Subversion repository. The JSON output can be, for example, re-injected into the BioVel refinement workflow. In a follow-up, the facility could be enhanced by using a Lucene-based index to speed up the export function.

*Code repository* - http://dev.e-taxonomy.eu/svn/trunk/cdmlib/

*Demo* - http://dev.e-taxonomy.eu/cdmserver/chenopodiumPilot/occurrence_catalogue.json?query=95402484-06cc-4284-a84c-193be51489ea&pageNumber=0&pageSize=10

**iPython notebook/Taverna**

*Members* - Youri Lammers,Ross Mounce, Aleksandra Pawlik and Alan Williams

*Accomplishments* - iPython notebook (Perez and Granger 2007) provides an interactive computational environment within a web browser in which users can write and execute code written in the Python programming language. This code may be combined with text, mathematical and statistical calculations, production of plots and HTML display to produce shareable and re-usable notebooks. The notebooks can be shared on the iPython Notebook Viewer. Taverna (Wolstencroft et al. 2013) provides a suite of tools for workflow design, editing and execution. This includes the Taverna Workbench, the main creation tool for workflows. The workflows allow the coordination of services, including RESTful or SOAP web services, R scripts and command line tools. Taverna Server enables you to set up a dedicated server for executing workflows remotely, and it can be accessed by a WSDL or a REST API. One of the deliverables of the BioVeL project (Vicario et al. 2011) is a workflow “player” that can be embedded in HTML, exposing the functionality of a given Taverna workflow within a web page. This breakout group sought to combine these two tools, such that iPython notebook users are able to submit data assembled during their analysis to a Taverna workflow embedded in a player and retrieving the results. The participants succeeded in this, resulting in a more integrated, powerful research environment that exposes remote HPC resources (such as BioVeL services) to iPython notebook users. The results are available for easy installation as the Pypi package *tavernaPlayerClient*.

*Code repository* - https://github.com/myGrid/DataHackLeiden

*Demo* - http://nbviewer.ipython.org/urls/raw.githubusercontent.com/myGrid/DataHackLeiden/alan/Player_example.ipynb?create=1

**BioVeL/NeXML services**

*Members* - Bachir Balech, Christian Brenninkmeijer, Hannes Hettling and Rutger Vos

*Accomplishments* - Biodiversity-oriented phylogenetics workflows usually involve various software tools connected in series that consume and produce different types of data. NeXML is an XML standard that supports the representation of (among others) taxa, character-state matrices, phylogenetic trees and semantic annotations within one single document and is therefore specifically tailored to ease the interplay of different tools in phylogenetic analysis (Vos et al. 2012). Since XML documents are generally intended to be handled by software rather than by users directly, a software tool to easily manipulate NeXML files appears desirable. To this end, the scope of this task group was to develop software that can i) construct NeXML documents from data encoded in commonly-used phylogenetic file formats or add metadata to an existing NeXML document (*NeXML merger*), and ii) extract information defined by the user from a given NeXML file (*NeXML extractor*). To make the NeXML merger and extractor tools easily accessible for the biodiversity research community and to enable their integration into existing workflows, they are implemented as RESTful web services hosted by Naturalis Biodiversity Center and made available in the BiodiversityCatalogue (https://www.biodiversitycatalogue.org/services/70). Preliminary tests of the NeXML merger and extractor have been conducted using data inputs and outputs used by the phylogenetic service set of BioVeL (Vicario et al. 2012) (https://www.biodiversitycatalogue.org/services/31/service_endpoint); NeXML extractor output has also been visualised as a phylogenetic tree with its taxon-associated metadata by implementing an ITOL (http://itol.embl.de/) tool wrapper within a Taverna workflow.

*Code repository* - https://github.com/naturalis/biovel-nbc

*Demo* - http://biovel.naturalis.nl/biovel?service=NeXMLExtractor&nexml=http://bit.ly/1mR11Yz&object=Trees

## Conclusions

The Biodiversity Data Enrichment Hackathon revealed how many possible applications for biodiversity data there are and how quickly solutions can be put together when the normal constraints to collaboration are broken down for a week. Biodiversity data was mobilised from its silos and enriched with meaningful links to related resources, such as links from taxon names to taxon concept URIs; links from described habitats to environment ontologies; links from character traits to trait ontologies; links from species treatments to relevant images, publications and specimens. The workflow tools and data publishing platforms that operate on such enriched data were enhanced to provide greater interoperability and data integration functionality.

The tangible outcomes of the hackathon are finding sustainable homes in the appropriate code bases (e.g. the code bases for the CDM platform, the Plazi server, the BHL server) as well as registries and repositories (e.g. the BiodiversityCatalogue, the Pypi index, the NCBO BioPortal), or form the basis of proofs-of-concept for further development, scientific publications and project proposals. The main intangible outcomes of the event are the further fostering of a community of experts in biodiversity informatics and the strengthened human links between research projects and institutions.

The event also demonstrated the ongoing need for data normalisation and integration, e.g. through the application of ontologies, and the opportunities for innovative research that such integration will afford. Mobilising biodiversity knowledge from their silos in the heritage literature and natural history collections will require tackling numerous factual, technical, economic, and sociological factors; as well as putative or real legal barriers, in particular, copyright and database protection rights. In the process, duplications and redundancies will be uncovered where, for instance, data, information and knowledge on taxonomic names are spread out over multiple institutions in the form of databases, catalogues and lists. Integration will further foster synergies among different disciplines and communities, in some cases encouraging specialisations that can be federated by others, and in other cases allowing rapid project-based development.

## Figures and Tables

**Table 1.** Themes, Breakout groups and Aims

**MOBILISING HERITAGE BIODIVERSITY KNOWLEDGE**	
Biodiversity data analytics	Extract statistical data about specimens for visualisation in a dashboard.
OCR correction	Provide a simple interface for interactively editing of OCR’d text, as well as tools to track the edits, to provide feedback to improve the OCR.
Open access images	Liberate and showcase openly-licensed (e.g. CC-BY) images from journal article PDFs and republish on image sharing social media sites. Find images of phylogenetic trees for data re-extraction from the image.
**FORMALISING AND LINKING CONCEPTS**	
Trait ontology	Extract and ontologise plant trait data from digitized Floras.
SWeDe	Produce a standard for describing scientific web services.
Specimen links	Link together name and specimen data, especially from Floras. Link specimen citations in “Literature” to specimens from Kew, Brussels and Edinburgh. Proof of concept and requirements gathering for Taxonomic MindMapper.
**SERVICE PLATFORMS**	
EDIT Platform Common Data Model API	Develop a web service to extract occurrences out of CDM instances (EDIT platform).
iPython notebook/Taverna	Access Taverna workflows from within iPython notebook.
BioVeL/NeXML services	Deliver RESTful services to merge and query phylogenetic data and metadata.
